# Radiological assessment of dissected cervical lymph nodes in level III affected by the area of supraomohyoid neck dissection

**DOI:** 10.1093/dmfr/twaf065

**Published:** 2025-09-12

**Authors:** Yohei Takeshita, Yoshio Ohyama, Soichiro Ibaragi, Yuki Matsushita, R Shane Tubbs, Norio Kitagawa, Toshiyuki Kawazu, Miki Hisatomi, Shunsuke Okada, Mamiko Fujikura, Yoshinobu Yanagi, Joe Iwanaga

**Affiliations:** Department of Oral and Maxillofacial Radiology, Faculty of Medicine, Dentistry and Pharmaceutical Sciences, Okayama University, Okayama, 700-8558, Japan; Clinical Anatomy Research Association in Oral and Maxillofacial Surgery, Fukuoka, 830-0011, Japan; Clinical Anatomy Research Association in Oral and Maxillofacial Surgery, Fukuoka, 830-0011, Japan; Oral and Maxillofacial Surgery, Shizuoka City Shizuoka Hospital, Shizuoka, 420-8630, Japan; Clinical Anatomy Research Association in Oral and Maxillofacial Surgery, Fukuoka, 830-0011, Japan; Department of Oral and Maxillofacial Surgery, Faculty of Medicine, Dentistry and Pharmaceutical Sciences, Okayama University, Okayama,700-8558, Japan; Clinical Anatomy Research Association in Oral and Maxillofacial Surgery, Fukuoka, 830-0011, Japan; Department of Skeletal Development and Regenerative Biology, Nagasaki University Graduate School of Biomedical Sciences, Nagasaki, 852-8588, Japan; Clinical Anatomy Research Association in Oral and Maxillofacial Surgery, Fukuoka, 830-0011, Japan; Department of Neurosurgery, Tulane Center for Clinical Neurosciences, Tulane University School of Medicine, New Orleans, LA, 70112, United States; Department of Neurology, Tulane Center for Clinical Neurosciences, Tulane University School of Medicine, New Orleans, LA, 70112, United States; Department of Structural & Cellular Biology, Tulane University School of Medicine, New Orleans, LA, 70112, United States; Department of Neurosurgery and Ochsner Neuroscience Institute, Ochsner Health System, New Orleans, LA, 70112, United States; Department of Anatomical Sciences, St George’s University, St George’s, Grenada; Department of Surgery, Tulane University School of Medicine, New Orleans, LA, 70112, United States; University of Queensland, Brisbane, 4072, Australia; Clinical Anatomy Research Association in Oral and Maxillofacial Surgery, Fukuoka, 830-0011, Japan; Department of Oral and Maxillofacial Anatomy, Graduate School of Medical and Dental Sciences, Institute of Science Tokyo, Tokyo, 113-8510, Japan; Department of Oral and Maxillofacial Radiology, Faculty of Medicine, Dentistry and Pharmaceutical Sciences, Okayama University, Okayama, 700-8558, Japan; Department of Oral and Maxillofacial Radiology, Okayama University Hospital, Okayama, 700-8558, Japan; Department of Oral and Maxillofacial Radiology, Okayama University Hospital, Okayama, 700-8558, Japan; Department of Oral and Maxillofacial Radiology, Okayama University Hospital, Okayama, 700-8558, Japan; Department of Oral and Maxillofacial Radiology, Faculty of Medicine, Dentistry and Pharmaceutical Sciences, Okayama University, Okayama, 700-8558, Japan; Clinical Anatomy Research Association in Oral and Maxillofacial Surgery, Fukuoka, 830-0011, Japan; Department of Neurosurgery, Tulane Center for Clinical Neurosciences, Tulane University School of Medicine, New Orleans, LA, 70112, United States; Department of Neurology, Tulane Center for Clinical Neurosciences, Tulane University School of Medicine, New Orleans, LA, 70112, United States; Department of Structural & Cellular Biology, Tulane University School of Medicine, New Orleans, LA, 70112, United States; Department of Neurosurgery and Ochsner Neuroscience Institute, Ochsner Health System, New Orleans, LA, 70112, United States; Dental and Oral Medical Center, Kurume University School of Medicine, Fukuoka, 830-0011, Japan; Division of Gross and Clinical Anatomy, Department of Anatomy, Kurume University School of Medicine, Fukuoka, 830-0011, Japan

**Keywords:** omohyoid muscle, CT, neck dissection, cervical lymph nodes, cancer

## Abstract

**Objectives:**

To compare the number of dissected cervical lymph nodes in the anatomical level III with that in supraomohyoid neck dissection (SOHND) level III affected by the anatomical relationship between the omohyoid muscle and cricoid cartilage using contrast-enhanced CT (CE-CT) images to assess the validity of the current SOHND.

**Methods:**

CE-CT images of the patients who suffered from malignant tumours in the oral and maxillofacial regions were reviewed. The number of cervical lymph nodes both in the anatomical level III (area between the centre of the inferior border of the hyoid bone [HB] and the inferior border of the cricoid cartilage [CC]) and SOHND level III (area between HB and the intersection of the omohyoid muscle and internal jugular vein [OM-IJ]) were recorded, respectively.

**Results:**

The rate of patients whose number of lymph nodes in level III was affected by the positional relationship between the OM-IJ and CC was almost equal in males and females. As for the patients with OM-IJ below the CC, the number of lymph nodes in SOHND level III increased from that of anatomical level III. Females showed significantly higher values than males (*P *< .05). Meanwhile, for patients with OM-IJ at or above the CC, the number of lymph nodes in SOHND level III decreased from that of anatomical level III.

**Conclusions:**

The number of dissected cervical lymph nodes differed between the SOHND dissection area and levels I, II, and III. In most cases, SOHND dissects more cervical lymph nodes, especially in female patients.

## Introduction

A supraomohyoid neck dissection (SOHND) is a surgical procedure that removes the tissue on and above the omohyoid muscle en bloc and is classed as a prophylactic selective neck dissection (SND) to treat patients with clinically node-negative.^1–5^ A SOHND was first described by Byers in his report of 967 cases.^1^ It is indicated for oral cancer or oropharyngeal cancer with no preoperative evidence of neck lymph node metastases. Generally, the cervical lymph nodes in the levels I, II, and III are removed by SOHND.^6^^,^^7^

In the levels of cervical lymph nodes, the superior and inferior borders of level III are anatomically defined as “horizontal plane defined by the inferior body of hyoid” and “horizontal plane defined by the inferior border of the cricoid cartilage (CC),”^8^ which is not directly correlated with the omohyoid muscle. It is assumed that the positional relationship between the omohyoid muscle and the cricoid cartilage might cause an excessive or insufficient dissection area. The positional relationship between the omohyoid muscle and the cricoid cartilage was investigated in the previous study.^9^ The intersection of the omohyoid muscle and internal jugular vein (OM-IJ) was located around the seventh cervical vertebra to the first thoracic vertebra. In contrast, CC was located around the sixth to seventh cervical vertebrae. Both sexes tended to have lower OM-IJ than CC, and females had significantly lower OM-IJ than males. Second, the number of males with the OM-IJ in level III and females with the OM-IJ in level IV were significantly larger, and males with the OM-IJ in level IV and females with the OM-IJ in level III were significantly smaller. Although many surgeons consider the SOHND to be the procedure for removing lymph nodes in levels I, II, and III,^7^^,^^10^ the anatomical evidence has shown that the dissection area of the SOHND and levels I, II, and III are inconsistent, especially in female patients.^9^ We hypothesized that the number of the removed cervical lymph nodes in level III during SOHND may differ from the actual number of cervical lymph nodes present in this level, potentially leading to different outcomes. When OM-IJ is located at or above the level of the CC, lymph nodes located in the gap between SOHND level III and anatomical level III could potentially represent undissected metastatic nodes. On the other hand, when the OM-IJ is located below the CC, it may be appropriate to narrow the dissection area to reduce the number of lymph nodes removed, given that SOHND is performed in clinically node-negative patients and with consideration for preserving quality of life.

This study aimed to investigate how the difference between the level III inferior border and OM-IJ influences the number of cervical lymph nodes dissected during SOHND, focusing on the anatomical relationship between the omohyoid muscle and cricoid cartilage using contrast-enhanced CT (CE-CT) images to assess the validity of the current SOHND.

## Methods

### Study subjects

We retrospectively reviewed CE-CT images of the head and neck region in Japanese patients referred to our institution between January 2019 and December 2021. All patients included in this study had a history of malignant tumours in the oral and maxillofacial region. CE-CT images taken at the patient’s initial visit or before the operation were included. The number of cervical lymph nodes located in level III, based on the intersection of the omohyoid muscle and internal jugular vein (OM-IJ) and CC, was recorded, respectively. The sex, age, body height, and weight of each patient were also recorded.

### Image preparation

CE-CT images were taken with 4 types of CT (Aquilion ONE: Canon Medical Systems Corporation, Tochigi, Japan; Aquilion Precision: Canon Medical Systems Corporation; Discovery CT750 HD: GE Healthcare, Milwaukee, WI, USA; SOMATOM Definition Flash: Siemens AG, Munich, Germany). The CT images had 3 slice thicknesses: 0.8, 1.00, and 1.25 mm. Axial images of the nonpathological were used for accurate measurements.

### Measurements

**Figure 1.**
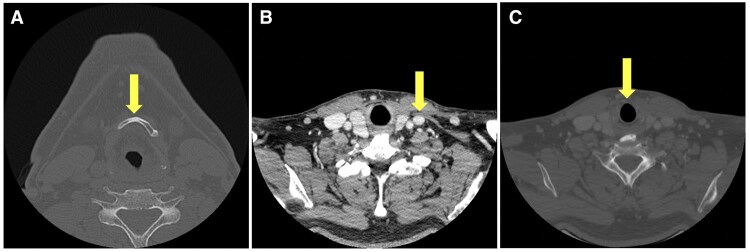
The points of measurement of anatomical structures in the slice of contrast-enhanced CT (CE-CT) images. (A) Centre of the inferior border of the hyoid bone (HB). (B) Intersection of the omohyoid muscle and internal jugular vein (OM-IJ). (C) Inferior border of cricoid cartilage (CC). The OM-IJ was identified using CE-CT images with soft tissue windows, and the other 2 anatomical structures (HB and CC) were identified with the bone windows.

**Figure 2.**
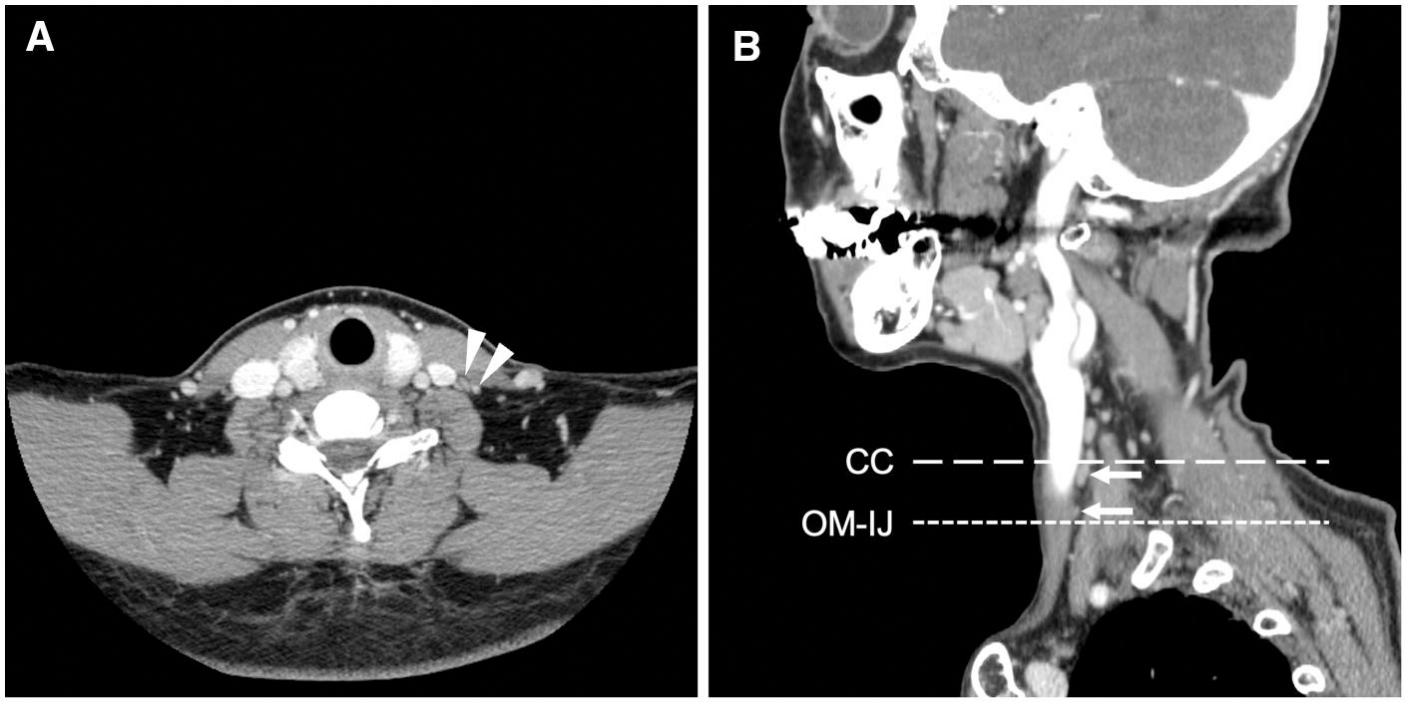
Example of axial and sagittal image of contrast-enhanced CT image with soft tissue windows. (A) The cervical lymph nodes in level III were observed (white arrowheads). (B) The cervical lymph nodes in the gap between the intersection of the omohyoid muscle and the internal jugular vein (OM-IJ) and the inferior border of the cricoid cartilage (CC) were observed (white arrows).

The reference points used in this study followed the previous studies by Takeshita et al.^9^ The slices of the CE-CT images corresponding to the following positions of anatomical structures were recorded: centre of the inferior border of the hyoid bone (HB); the intersection of omohyoid muscle and internal jugular vein (OM-IJ); CC. The OM-IJ was identified using CE-CT images with soft tissue windows. The HB and CC were identified with bone windows (Figure 1). Subsequently, the number of cervical lymph nodes both in the anatomical level III (area between HB and CC) and SOHND level III (area between HB and OM-IJ) was recorded, respectively, using CE-CT images with soft tissue windows (Figure 2).

One oral and maxillofacial radiologist performed all the measurements of the CE-CT images. Measurements were taken twice over a one-month interval.

### Statistical analysis

Intraobserver agreement between the first and second measurements was measured using intraclass correlation coefficients and classed into 5 grades: poor agreement (<0.20), fair agreement (0.21-0.40), moderate agreement (0.41-0.60), good agreement (0.61-0.80), excellent agreement (0.81-1.00). The significance of differences among measurements was measured by Pearson’s chi-square test and the unpaired *t*-test using Stata (version 18.0; StataCorp LLC, TX, USA). *P *< .05 was considered statistically significant.

The present study protocol was approved by the Okayama University Ethics Committee (approval No. 2203-007), and the study was performed in accordance with the requirements of the Declaration of Helsinki (64th WMA General Assembly, Fortaleza, Brazil, October 2013). Informed consent was obtained from all patients for inclusion in the study.

## Results

Fifty-five patients (25 males and 30 females) with a history of malignant tumours in the oral and maxillofacial region were evaluated. The nonpathological sides of 32 right-side necks and 23 left-side necks were included. The mean age was 69.0 years (range 30-93); the mean age of males was 68.6 years (range 37-89) and of females was 69.3 years (range 30-93). The mean height and weight were 157.3 cm (range 135.5-180.0 cm) and 51.1 kg (range 29.4-82.5 kg), respectively. In the males, the corresponding values were 165.0 cm (range 154.5-180.0 cm) and 57.2 kg (range 43.9-82.5 kg); in the females, they were 150.9 cm (range 135.5-168.1 cm) and 46.0 kg (range 29.4-62.1 kg), respectively (Table 1).

**Table 1.** Distribution of patients in this study.

	Total	Male	Female
No. of patients	55	25	30
Mean age (years)	69.0 (range 30-93)	68.6 (range 37-89)	69.3 (range 30-93)
Mean height (cm)	157.3 (range 135.5-180.0)	165.0 (range 154.5-180.0)	150.9 (range 135.5-168.1)
Mean weight (kg)	51.1 (range 29.4-82.5)	57.2 (range 43.9-82.5)	46.0 (range 29.4-62.1)

### Intraclass correlation coefficients

The intraclass correlation coefficients for the slice of CE-CT images of the HB, OM-IJ, and CC were 0.999, 0.999, and 0.999, respectively. Those for the number of cervical lymph nodes in the anatomical level III and SOHND level III were 0.772 and 0.754, respectively. All intraclass correlation coefficients showed good to excellent agreement. The second measurements were used for subsequent analysis.

### Difference in the number of cervical lymph nodes between anatomical level III and SOHND level III

Among the 55 patients, 42 (76.4%) had a different number of cervical lymph nodes between anatomical level III and SOHND level III (ranging from −3 to 5), and 13 (23.6%) had the same number of cervical lymph nodes in both groups.

**Figure 3.**
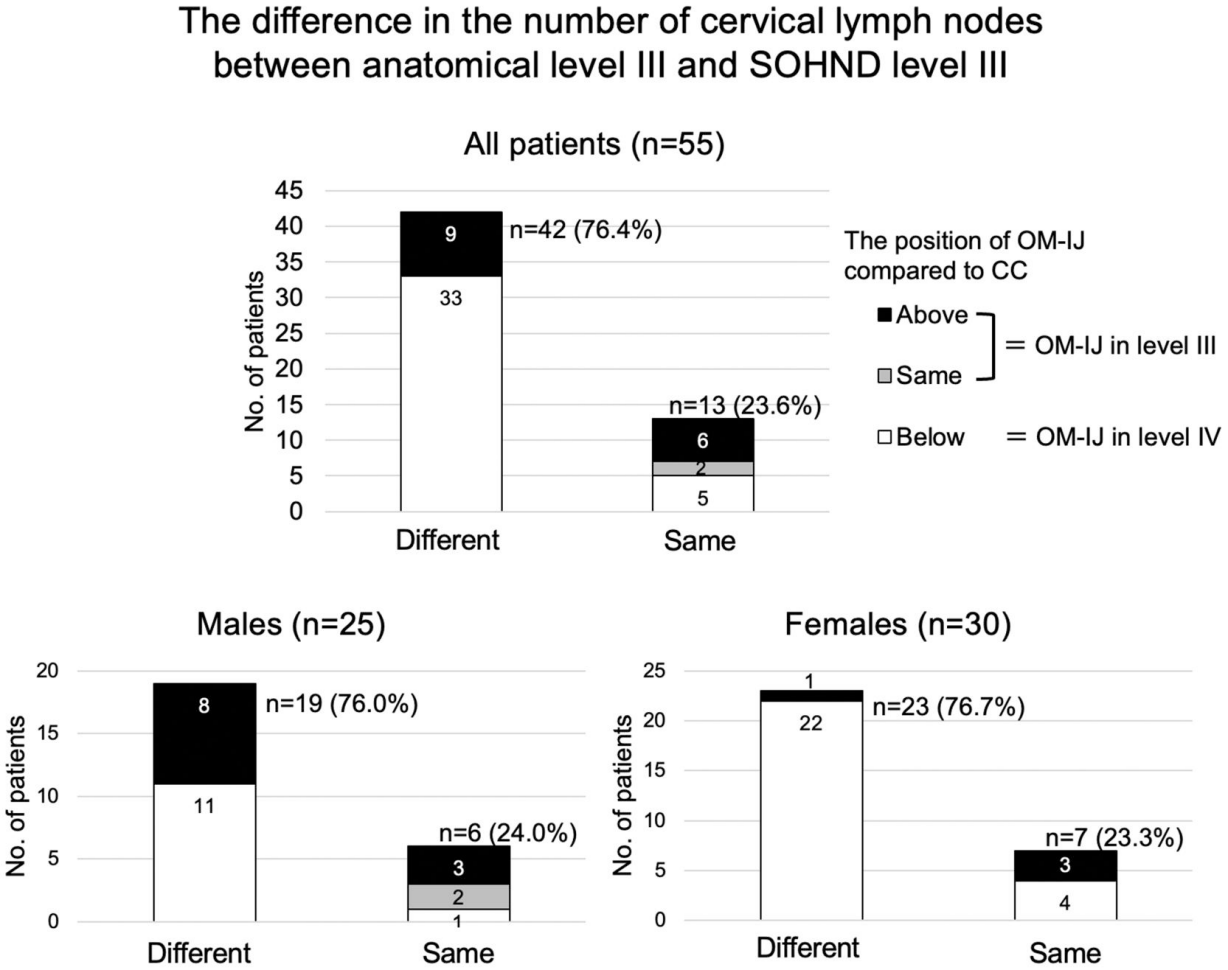
The difference in the number of cervical lymph nodes in level III. The rate of patients whose number of cervical lymph nodes in level III was affected by the positional relationship between the intersection of the omohyoid muscle and the internal jugular vein (OM-IJ) and the inferior border of the cricoid cartilage (CC) was almost equal in males and females, and there was no significant sex difference (*P *> .05).

Of the 25 males, 19 (76.0%) had different cervical lymph nodes between anatomical level III and SOHND level III, and 6 (24.0%) had the same number of cervical lymph nodes in both groups. Of the 30 females, 23 (76.7%) had different cervical lymph nodes between anatomical level III and SOHND level III, and 7 (23.3%) had the same number of cervical lymph nodes in both groups. There was no significant difference between the sexes (*P *> .05, Pearson’s chi-square test) (Figure 3).

### Positional relationship between OM-IJ and CC

**Figure 4.**
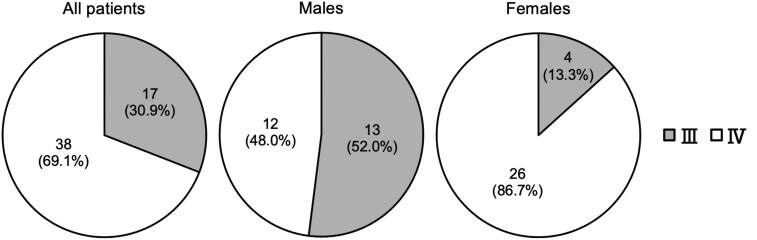
Distribution of the positional relationship between the intersection of the omohyoid muscle and the internal jugular vein (OM-IJ) and the inferior border of the cricoid cartilage (CC) in relation to the level of the lymph node regions of the neck. Males with OM-IJ in level III and females with OM-IJ in level IV were significantly larger, and males with OM-IJ in level IV and females with OM-IJ in level III were significantly smaller (*P *= .002).

Among the 55 patients, the OM-IJ was located below the CC, ie, OM-IJ was in level IV in 38 patients (69.1%), and OM-IJ was located at or above the level of the CC, ie, OM-IJ was in level III in 17 patients (30.9%). Of the 25 males, 12 (48.0%) had the OM-IJ in level IV and 13 (52.0%) in level III. Of the 30 females, 26 (86.7%) had the OM-IJ in level IV and 4 (13.3%) in level III. The number of males with the OM-IJ in level III and females with it in level IV were significantly larger, and males with the OM-IJ in level IV and females with it in level III were significantly smaller (*P *= .002, Pearson’s chi-square test) (Figure 4).

### Number of cervical lymph nodes in SOHND level III with OM-IJ below the CC (OM-IJ within level IV)

Out of 38 patients with OM-IJ in level IV, the number of cervical lymph nodes in SOHND level III was larger than in anatomical level III in 33 patients (11 males and 22 females). The mean difference and rate of change in the number of cervical lymph nodes between SOHND level III and anatomical level III were 2.18 (male: 1.45; female: 2.55) and 49.3% (male: 28.6%; female: 62.2%), respectively. Females showed significantly larger values in both points (difference: *P *= .01; rate of change: *P *= .03, unpaired *t*-test). The number of cervical lymph nodes was the same in both SOHND level III and anatomical level III in the remaining 5 patients (male: 1; female: 4) (Table 2).

**Table 2.** Difference in the number of cervical lymph nodes in SOHND level III when the OM-IJ is located below the CC (ie, OM-IJ within level IV).

	Total	Male	Female	
Number of patients	33	11	22	
Mean difference (number)	2.18	1.45	2.55	*P = .01
Mean rate of change (%)	49.3	28.6	62.2	*P = .03

### Number of cervical lymph nodes in SOHND level III with OM-IJ at or above the CC (OM-IJ within level III)

Out of 17 patients with OM-IJ in level III, the number of cervical lymph nodes in SOHND level III was smaller than in anatomical level III in 9 patients (male: 8; female: 1). The mean difference and rate of change in the number of the cervical lymph between SOHND level III and anatomical level III was −1.07 (male: −1.63; female: −3.00) and −32.7% (male: −28.9%; female: −75.0%), respectively. The significant sex difference could not be analysed because of the small number of patients. The number of cervical lymph nodes was the same in both SOHND level III and anatomical level III in the remaining 8 patients (male: 5; female: 3) (Table 3).

**Table 3.** Difference in the number of cervical lymph nodes in SOHND level III when the OM-IJ is located at or above the CC (ie, OM-IJ within level III).

	Total	Male	Female
Number of patients	9	8	1
Mean difference (number)	−1.07	−1.63	−3.00
Mean rate of change (%)	−32.7	−28.9	−75.0

## Discussion

It is well recognized that the primary regions of metastatic lymph nodes of oral and oropharynx cancer are at levels I and II.^11^ An SND is accepted as a surgical procedure to treat the metastatic cervical lymph nodes in various stages. An SOHND especially dissects the cervical lymph nodes in levels I, II, and III and is often indicated for patients with a clinically metastasis-free neck (no metastatic lymph nodes).^1–3^^,^^12–15^ Reliable anatomical landmarks are essential to standardize the neck dissection procedure, as the positions of these structures can influence the extent of the dissection. To assess the validity of the current SOHND, we investigated the number of cervical lymph nodes in the dissection area of SOHND, focusing on the anatomical relationship between the omohyoid muscle and cricoid cartilage with CE-CT images based on the previous study.^9^

The number of cervical lymph nodes in anatomical level III and SOHND level III differed in 76.4% of patients (76.0% in males and 76.7% in females). According to Takeshita et al,^9^ OM-IJ tends to be located below the CC in both sexes.

The number of cervical lymph nodes in the SOHND level III increased when the OM-IJ was within level IV and decreased when the OM-IJ was within level III. As the OM-IJ is more likely to be located within level IV, the SOHND procedure often invades level IV, not only levels I, II, and III. As the OM-IJ is more often located within level III than level IV in male patients in the previous study, the dissection area of SOHND in male patients is considered less invasive, and the influence on the number of dissected cervical lymph nodes is relatively low. On the other hand, especially in female patients, the OM-IJ was more frequently located within level IV in the previous study; SOHND in female patients seems more invasive than it is supposed to be. Albuja-Cruz et al^16^ reported that the recurrence of papillary thyroid cancer after modified radical neck dissection was not correlated with the number of lymph nodes removed, and they suggested that efforts to maximize the number of removed nodes might be unnecessary. On the other hand, some studies reported that the incidence of metastatic cervical lymph nodes in level III ranged from 9.2% to 21.9% in clinically metastasis-free necks of patients with oral squamous cell carcinoma who underwent SOHND.^17^^,^^18^ Given our focus on oral cancer patients and the number of lymph nodes within the dissection area of SOHND, further discussion and clarification are needed among surgeons performing SOHND and radiologists.

The results of this study raise concerns about post-SOHND metastatic lymph nodes located outside SOHND level III but within anatomical level III. Although distinguishing between these 2 levels in patients after SOHND is challenging—since the omohyoid muscle is generally removed along with surrounding tissues—lymph nodes in the gap between SOHND level III and anatomical level III could potentially be undissected metastatic nodes, especially when the OM-IJ is positioned above the CC. Once additional anatomical and clinical evidence is gathered, the discrepancies arising from this gap should be addressed and discussed among experts in the field. The advantage of radiological examination is that it allows preoperative assessment of anatomical risk without being invasive, potentially improving the procedure and patients’ quality of life.

In conclusion, this study clarified that the number of cervical lymph nodes in SOHND level III and anatomical level III was affected by the relationship between the OM-IJ and CC, especially in female patients. Further investigation is required to determine whether the difference in the dissection area influences the actual number of cervical lymph nodes removed and patient prognosis. It has been suggested that, in some cases, the dissection area may warrant modification. Future studies should examine the incidence of lymph node metastasis at the border between levels III and IV in patients undergoing SOHND.

### Limitation

Racial or ethnic differences may have influenced the results because all patients included in this study were Japanese. We used 3 different slice thicknesses of the CT image. The patients’ positions for the CT scan might not have been entirely the same, so there could have been small errors, especially in the actual measurements. A single observer evaluated the images, and there was lack of surgical or pathological evaluations.
